# American Dental Association

**Published:** 1862-09

**Authors:** 


### Editorial.

### AMERICAN DENTAL ASSOCIATION.

This body met in the city of Cleveland on the last Tuesday of July, and was quite as well attended as we anticipated. Eleven dental associations were represented, and three of these for the first time.

The feeling that was manifested in regard to this movement by the members was very gratifying. It seems to be the settled purpose of all the members, at least of those present, that this association shall accomplish that for which it was established, viz.: the development of the science of the dental profession, and encouraging the formation of local associations throughout the country. These are the two prominent objects of this organization. It does not, neither do any of its members desire to have it come in conflict with any organization or association of the profession. We were somewhat surprised to find some entertaining the idea that it was originated for the purpose of breaking down, or rather of taking the place of the American Dental Convention. No one acquainted with the work it proposes to do would for a moment entertain such an opinion.

There were a number of essays read, nearly all of which were highly interesting and instructive, and must have been prepared at the expense of much time, labor and research. By such productions as these can be seen, perhaps more fully than in anything else, the real progress of the profession, and the basis upon which it rests. The science is the foundation, and the art the superstructure.

It was refreshing to see the facility and harmony with which the work was accomplished. The committees all have their appropriate work, and know just what they have to do. Every member knows what is expected of each committee, and looks forward to the reports with interest, and lives in the anticipation of something valuable being brought forth.

In regard to the publication of the proceedings and essays in the journals of the profession, there was some discussion, and at first some diversity of opinion; but upon a full examination of the matter, all entertained, decidedly, the opinion that they should be excluded from the journals, and published in a volume of transactions, for the use of the members and those specially interested.

The argument by which this conclusion was attained we do not now propose to present; but will simply state that, for the most part, it was drawn from the influence such a course would have upon the formation of local societies. All members who pay dues will be entitled to a copy of the transactions, and all members of associations represented will be entitled to the transactions, by paying the amount fixed upon by the Association.

Of this action of the Association nobody has a right to complain; the members certainly have not, and those who are not members, and are not represented, and refuse to place themselves in that attitude, have nothing to say in the matter. It has been suggested that perhaps injustice might be done to the authors of essays, by publishing or using them in a manner contrary to their choice. When any one writes an essay for presentation to a public organized body, that production becomes the property of the association, to be disposed of as it sees fit, and the author has no more to do with it than he has with "any other man's" property, except by special reservation.

The establishment of this association we consider eminently a success, and hope it will receive the aid and encouragement of every one who has the good of the profession at heart. T.

